# Toward High-Peak-to-Valley-Ratio Graphene Resonant Tunneling Diodes

**DOI:** 10.1021/acs.nanolett.3c02281

**Published:** 2023-09-05

**Authors:** Zihao Zhang, Baoqing Zhang, Yiming Wang, Mingyang Wang, Yifei Zhang, Hu Li, Jiawei Zhang, Aimin Song

**Affiliations:** † Shandong Technology Center of Nanodevices and Integration, School of Microelectronics, Shandong University, Jinan 250100, China; ‡ Suzhou Research Institute, Shandong University, Suzhou 215123, China; § Department of Electrical and Electronic Engineering, University of Manchester, Manchester M13 9PL, United Kingdom

**Keywords:** Graphene, Resonant tunneling diode, Negative differential resistance, van der Waals heterostructure

## Abstract

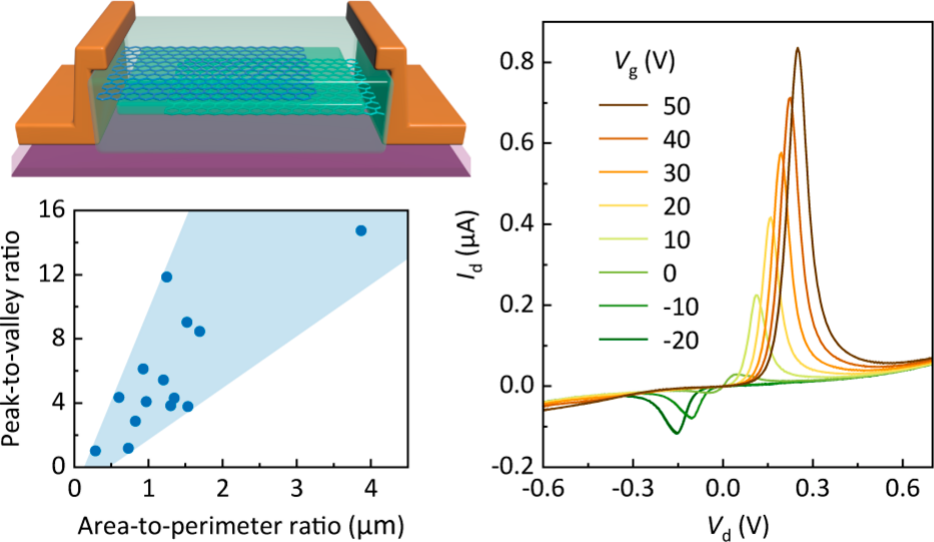

The resonant tunneling diode (RTD) is one of the very few room-temperature-operating quantum devices to date that is able to exhibit negative differential resistance. However, the reported key figure of merit, the current peak-to-valley ratio (PVR), of graphene RTDs has been up to only 3.9 at room temperature thus far. This remains very puzzling, given the atomically flat interfaces of the 2D materials. By varying the active area and perimeter of RTDs based on a graphene/hexagonal boron nitride/graphene heterostructure, we discovered that the edge doping can play a dominant role in determining the resonant tunneling, and a large area-to-perimeter ratio is necessary to obtain a high PVR. The understanding enables establishing a novel design rule and results in a PVR of 14.9, which is at least a factor of 3.8 higher than previously reported graphene RTDs. Furthermore, a theory is developed allowing extraction of the edge doping depth for the first time.

Van der Waals (vdW) heterostructures based on two-dimensional (2D) materials have attracted considerable attention owing to their new functionalities originating from the interface between dissimilar materials or from the stacking of crystals with different electronic structures.^1^ Recently, numerous studies on vdW heterostructures have been conducted, such as interlayer excitons,^2−5^ superconductivity,^6,7^ ferromagnetism,^8,9^ and photodetectors.^10−12^ For applications in high frequency electronics,^13^ multivalued logic gates,^14−17^ and memory devices,^18^ resonant tunneling diodes (RTDs) have drawn significant attention owing to their peculiar characteristics of negative differential resistance (NDR). RTDs are based on the quantum tunneling effect and operate at room temperature. Unlike conventional III–V RTDs, which require at least two barriers, RTDs based on vertically stacked 2D materials only need a single barrier to generate a pronounced room-temperature quantum effect. They can have much higher device speed because of the absence of the carrier dwell time in the central quantum well.^19^ The first 2D RTD was theoretically proposed and experimentally demonstrated in 2013, which was fabricated using a monolayer graphene/hexagonal boron nitride (h-BN)/monolayer graphene heterostructure.^19^ Its peak-to-valley ratio (PVR) of the current, which is a widely used metric for measuring NDR performance, was 1.8 at 300 K and 3.8 at 7 K. Subsequently, several graphene RTDs (GRTDs) were reported from 2014 to 2021,^13,18,20−23^ among which the highest PVRs were reportedly 3.9 at 300 K and 5.8 at 1.5 K, achieved in a bilayer graphene/WSe_2_/bilayer graphene heterostructure.^22^ From 2014 to 2022, several vertical NDR devices stacked by other 2D materials, including various combinations with MoS_2_, MoSe_2_, WSe_2_, SnSe_2_, InSe, and black phosphorus, were proposed and fabricated.^14−17,24−37^ Currently, the highest PVRs are 5.8 at 300 K and 10 at 50 K, achieved by a GeSe/HfS_2_ heterostructure reported in 2022.^17^ PVR is important for the applications of NDR devices. For example, in high-frequency applications, PVR determines the d.c.-to-RF conversion efficiency,^38^ and in multivalued applications, PVR determines the voltage range for accessing the middle state of the three states.^39^ The reported PVR values of graphene and all 2D RTDs in the literature are no more than 3.9 and 5.8, respectively, at room temperature thus far. This has been a very puzzling problem, given the atomically flat interfaces of the 2D materials used in 2D RTDs.

Although numerous advances have been made in 2D RTDs, only a few studies have focused on the mechanisms that determine the NDR performance. In RTDs, the tunneling current is determined by momentum and energy matching. To improve the momentum-matching conditions in GRTDs, the crystallographic orientation alignment of two graphene flakes is required. Originally, such an alignment was realized by manually rotating the graphene flakes under an optical microscope, and the alignment was verified by Raman spectroscopy.^13^ Later, a “tear and stack” technique was introduced to further improve the alignment precision.^21^ The alignment of the crystallographic orientations of the two graphene flakes determines the momentum conservation when tunneling occurs. However, there is a lack of research on the improvement of energy matching.

This study proposed that the energy matching can be disturbed by the edge doping of graphene, which, to the best of our knowledge, has not been studied in RTD devices. GRTDs with different geometric configurations were fabricated, and their geometry-dependent NDR performance was analyzed. By variation of the etching patterns of the GRTDs, the doping profile at the edge was found to disrupt the potential uniformity of graphene, thus limiting the current PVR. To further understand the mechanism, an analytical model was proposed, and a numerical simulation was performed, both of which agreed closely with the experimental data. Two parameters, the effective permeation depth (λ_eff_) and area-to-perimeter ratio (APR), were introduced to describe the permeation extent of edge doping. Next, a GRTD was fabricated with the understanding, and it achieved PVRs of 14.9 at 300 K and 23.9 at 9.6 K, both of which, to the best of our knowledge, are by far the highest values in 2D-material vertical NDR devices.

A structural schematic of the GRTD is shown in Figure 1a. The vertically stacked tunneling structure of monolayer graphene/h-BN/monolayer graphene was encapsulated by thick h-BN flakes. The barrier h-BN flake was composed of between three and five atomic layers, corresponding to 1–1.7 nm. A heavily p-doped Si wafer as the gate electrode, with thermally oxidized SiO_2_, was used as the substrate. The optical micrograph of a fabricated GRTD (device A) is shown in Figure 1b, and the output characteristics of device A under various gate voltages *V*_g_ are shown in Figure 1d (detailed device fabrication and characterization methods are demonstrated in section 1 of the Supporting Information).

**Figure 1:**
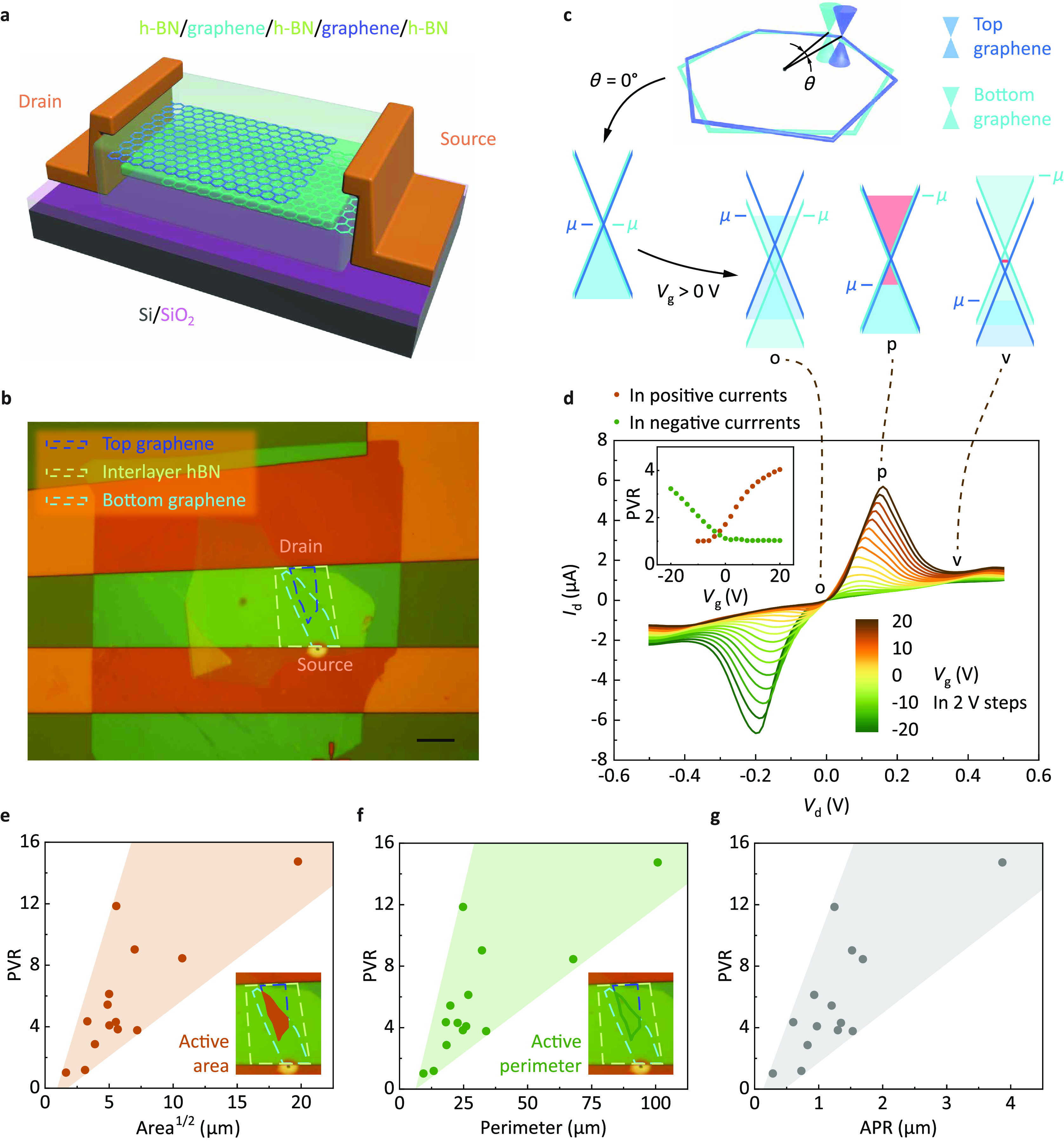
Schematics, electronic characteristics, and PVR distributions of GRTDs. (a) Structure schematic of GRTD. (b) Optical micrograph of a fabricated device; the scale bar is 10 μm. (c) Resonant tunneling schematic of two graphene electrodes separated by an h-BN tunnel barrier. (d) Output characteristics of the device in b under various gate voltages *V*_g_; the inset shows extracted PVR values as a function of *V*_g_. (e–g) PVR distributions of 14 devices in area (e), perimeter (f), and APR (g). Horizontal axes are unified into the dimension of length. PVR refers to the highest PVR with *V*_g_ ranging from −120 to 120 V. Area and perimeter refer to the active area and active perimeter measured from the overlap region of two graphene flakes. The insets of e and f show schematics demonstrating the measuring approaches for area and perimeter, respectively. All device measurements are performed at room temperature.

The working mechanism of GRTD can be explained with the help of the band diagram shown in Figure 1c, which corresponds to the output curve with *V*_g_ = 20 V in Figure 1d. A twist angle θ between the crystallographic orientations of two graphene flakes in real space leads to a separation of the Dirac cones of the two graphene flakes in reciprocal space. However, because the tear and stack technique was adopted, the twist angle could be minimized. Assuming θ = 0°, the Dirac cones could be horizontally aligned, thereby resulting in a perfect match of the momentum. Thus, only the vertical alignment (energy matching) must be considered. Without applying *V*_g_ and drain voltage *V*_d_, the graphene flakes encapsulated in h-BN were considered to be lightly doped. In this case, the Dirac cones were vertically aligned, but no tunneling current occurred. Note that applying only *V*_d_ can result in a low tunneling current and a mismatch in the energy band. Therefore, *V*_g_ was applied to draw a Dirac cone back (see point o in Figure 1c,d) owing to the quantum capacitance of graphene. At a certain *V*_d_ value (see point p), the two Dirac cones overlapped, and the highest tunneling current was achieved. With increasing *V*_d_, the tunneling current decreased owing to the vertical misalignment of the Dirac cones. The decreasing current with an increasing *V*_d_ is regarded as the NDR regime. The PVR, which is the figure of merit for NDR devices, is defined as the peak current divided by the valley current (see point v). A high PVR is essential for applications in high-frequency oscillation, amplification, and signal detection. However, most previous studies discussed the effects of different materials and transfer methods on the performance of 2D RTDs. Note that in this study, the device geometry also affected the electrical performance.

Although graphene is a zero-band gap material, its carrier density near the Dirac point is approximately zero. However, obtaining highly resistive graphene is challenging, because of unintentional inhomogeneous doping during the fabrication process. The Dirac points on a graphene flake spread spatially in terms of energy, thereby resulting in a high carrier concentration. Moreover, when a graphene flake is encapsulated in h-BN, the edge of the graphene flake is more likely to gather impurity atoms and contaminants because of the dangling bonds and poor coverage of the atomic steps. This results in a higher doping profile at the edge of the graphene flake. The resonant tunneling current of the GRTD is related to the dopant-induced energy distribution. Inhomogeneous doping broadens and suppresses the tunneling peak and finally decreases the PVR. Thus, the highly doped graphene along the perimeter most likely deteriorates the electrical performance of the GRTD.

To verify this assumption, 14 GRTDs with different geometric parameters, including area and perimeter, were fabricated on the Si substrate with 300-nm-thick SiO_2_ (detailed information on the devices is demonstrated in section 2 of the Supporting Information). All the devices were fabricated using the same techniques of mechanical exfoliation, dry transfer, and metal deposition. The highest PVR for each device was selected from PVRs with *V*_g_ ranging from −120 to 120 V. The active area and perimeter were measured from the overlapping region between the two graphene flakes, where the tunneling current occurred, using an optical microscope. The PVR values of the 14 devices as a function of the area and perimeter are shown in Figure 1e,f. Note that the PVR has positive correlations with both area and perimeter. Another geometric parameter, the area-to-perimeter ratio (APR), was obtained by dividing the area by the perimeter. As shown in Figure 1g, the PVR and APR also had a positive correlation. However, the area, perimeter, and APR of each device may all have a strong correlation. For example, for a circle of radius of *r*, the area π*r*^2^, perimeter 2π*r*, and APR *r*/2 all increase with increasing *r*.

To explore the determinant geometric factors, multiple etching processes were introduced to intentionally manipulate the area and perimeter of a single GRTD. Two devices were fabricated in this study. For the first GRTD (device B), the device was narrowed from the left and right sides by etching, which greatly reduced the area but less significantly reduced the perimeter; this was called the “area-etching” process (the area changed with etching). For the second GRTD (device C), the device was etched with many very fine grooves (100 nm wide), which greatly increased the perimeter but kept the area almost constant; this was called the “perimeter-etching” process (the perimeter changed with etching). The proposed etching concepts are illustrated in Figure 2a for “area etching” and Figure 2b for “perimeter etching”. Each etching process consists of four steps, namely, etchings #1–4. Optical micrographs of the two devices after each etching step are shown in Figure 2c,d. All of the PVR values were measured at *V*_g_ = 120 V. The PVR as a function of area, perimeter, and APR is shown in Figure 2e–g. The PVRs of both devices exhibited a linear relationship with area^–1^, perimeter^1^, and APR^–1^. However, they exhibited different slopes when the PVR was plotted as a function of area^–1^ and perimeter^1^. As shown in Figure 2f, the PVR of the “area-etching” device increased with the perimeter, whereas that of the “perimeter-etching” device decreased with the perimeter. As shown in Figure 2e, the PVRs of both the “area-etching” and “perimeter-etching” devices decreased with area^–1^; however, the latter exhibited a slope more than 40 times larger than that of the former (−150 μm^2^ to −3.2 μm^2^). As shown in Figure 2g, the PVRs for the two etching processes decreased with APR^–1^, and their slopes and intersections with a PVR = 1 were similar. This result suggests that the APR is the determinant geometric factor for device performance.

**Figure 2:**
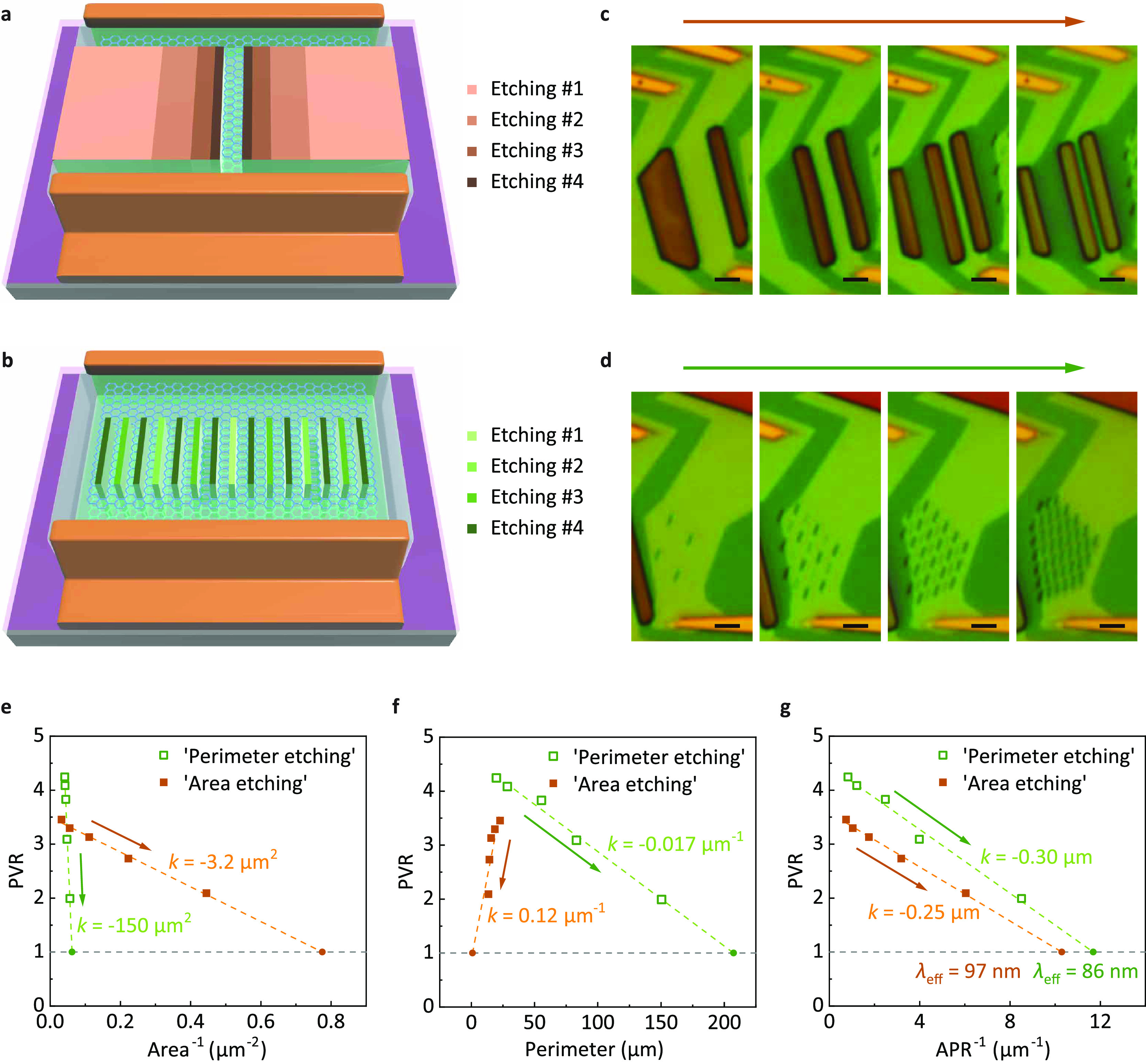
Variations in the PVR in two etching processes. (a,b) Schematics of “area etching” (a) and “perimeter etching” (b). Each etching process consists of four steps, namely etchings #1–4. (c,d) Optical micrographs of the “area-etching” device (device B; c) and “perimeter-etching” device (device C; d). From left to right, the photos are taken when etchings #1–4 are finished. The scale bars are 2 μm. (e–g) PVR during etching as a function of area (e), perimeter (f), and APR (g). Five data points are shown for each process, including the value measured before etching and after etchings #1–4. Arrows indicate the sequences of etching steps. The PVR is measured under the gate voltage *V*_g_ = 120 V. Area^–1^, perimeter^1^, and APR^–1^ exhibit a better linear relation with PVR. Here, orange, light green, and gray dashed lines indicate the linear fitting of the “area-etching” data, the linear fitting of the “perimeter-etching” data, and PVR = 1, respectively. *k* is the slope of the linear fitting, and λ_eff_ is the APR of the intersection of the linear fitting and PVR = 1.

Experimental results have demonstrated that there must be some edge effects that constrained the device performance. Although other possibilities cannot be ruled out, this study suggested that edge doping is the most plausible explanation. An analytical model of edge doping was established. As shown in Figure 3a, a graphene flake with an arbitrary shape can be treated as two parts: a heavily doped region close to the edge and a lightly doped region far from the edge. The heavily doped region distributes along the perimeter and has a doping depth of λ_eff_, and it has an area of Area_h_ ≈ Perimeter × λ_eff_. The left is the lightly doped region with an area of Area_l_ ≈ Area – Perimeter × λ_eff_. The tunneling current of a GRTD of an arbitrary shape can be treated as two parts, originating from the heavily doped region with a current density of *J*_h_ (*V*_d_) and from the lightly doped region with a current density of *J*_l_ (*V*_d_). Assuming that the voltage at the peak current is *V*_d,peak_ and that the voltage at the valley is *V*_d,valley_, the PVR is given by
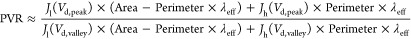
1

**Figure 3:**
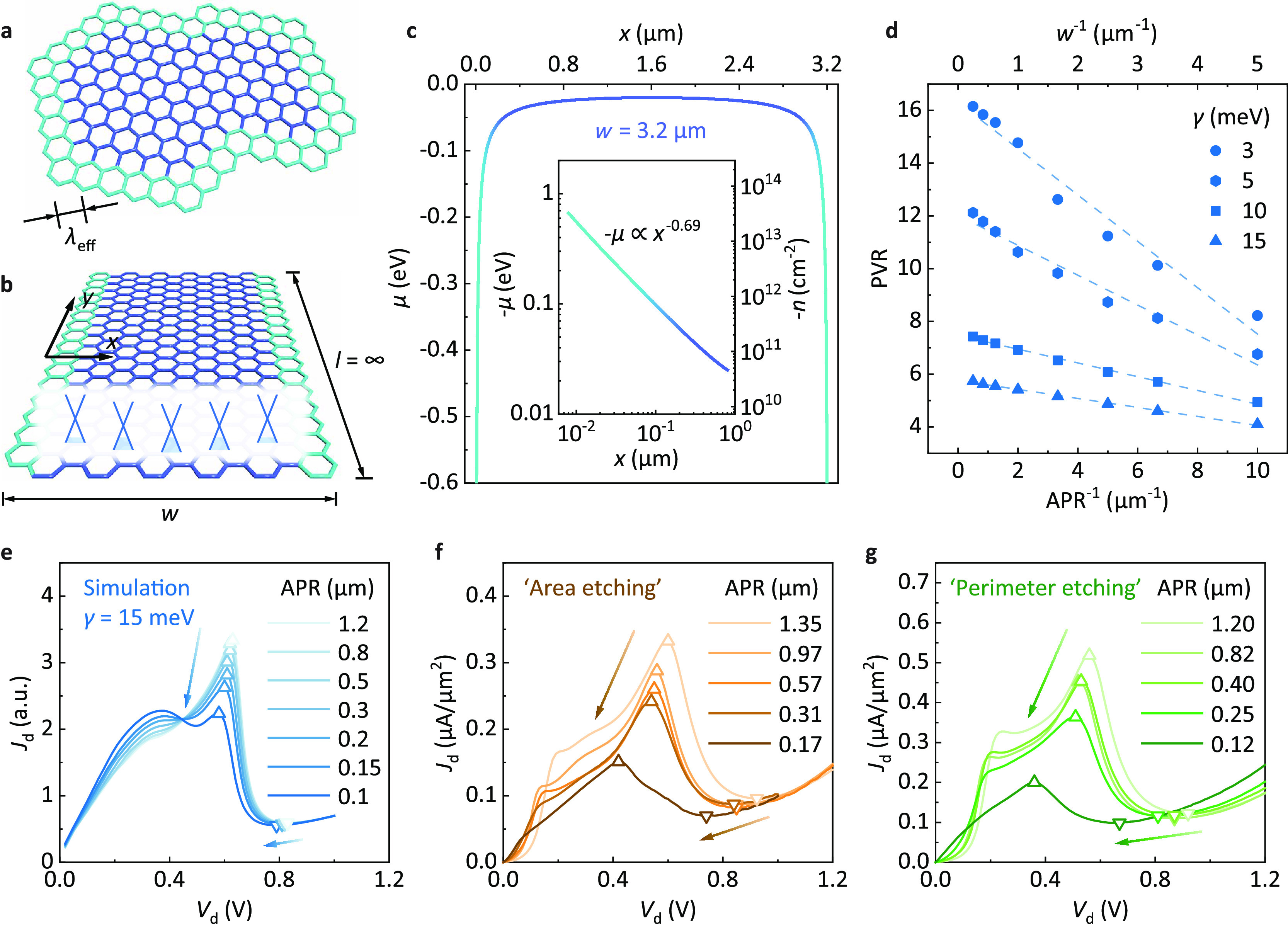
Mechanism of edge doping. (a) Edge doping schematic in a graphene flake of arbitrary shape. The cyan lattice indicates the heavily doped region, whereas the blue lattice indicates the lightly doped region. (b) Edge doping schematic in a graphene ribbon with a length *l* = ∞ along the *y* axis and width *w* along the *x* axis. (c) Simulation results of the distribution of the chemical potential μ along the *x* axis in a graphene ribbon with *w* = 3.2 μm. The inset is distributions of μ and the sheet carrier density *n* in double-logarithmic coordinates; μ and *n* are approximately proportional to a power function of *x* beside the left edge (*x* = 0). (d) Simulation results of the PVR of GRTD as a function of APR and *w*. Here, the edges of two graphene flakes coincide in location and have the same doping level, the gate voltage is *V*_g_ = 120 V, the broadening of electronic states γ is in the range of 3–15 meV, and the bottom axis is APR^–1^ to enable comparison with Figure 2g. (e–g) Simulation results (e), along with “area-etching” (f) and “perimeter-etching” (g) results for comparison, of output characteristics with various APRs under *V*_g_ = 120 V; here, γ = 15 meV. Arrows show the directions that peaks and valleys change along with decreasing APR.

Because of the heavy doping profile at the edge, the assumptions made are that, in the heavily doped region, the resonant tunneling condition is disturbed, and no NDR characteristic exists (PVR = 1). Thus, the peak current originating from resonant tunneling decreases to the value of the valley current, whereas the valley current originating from nonresonant tunneling remains unchanged, which implies *J*_h_ (*V*_d, peak_) ≈ *J*_h_ (*V*_d, valley_) ≈ *J*_l_ (*V*_d, valley_). The PVR can be described by
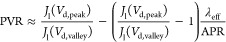
2

This result indicates that the PVR is linearly related to APR^–1^, which agrees closely with the experimental results shown in Figure 2g. Additionally, when PVR ≈ 1, APR = λ_eff_, which suggests that when the overlap region of two graphene flakes contains only the heavily doped region, the NDR characteristic disappears. Therefore, not only can λ_eff_ be called the effective permeation depth of edge doping but also it is a noteworthy indicator of RTD devices to evaluate their electrical performance. As shown in Figure 2g, the linear fitting indicates that the λ_eff_ values of the “area-etching” and “perimeter-etching” devices were 97 and 86 nm, respectively. To acquire an ultrahigh PVR, the requirement of APR ≫ λ_eff_ must be satisfied. The introduction of λ_eff_ provides another insight into the NDR characteristic in GRTDs.

Next, a numerical simulation was performed (detailed calculations and parameters are provided in section 3 of the Supporting Information). As shown in Figure 3b, by assuming an infinitely long graphene ribbon with a width of *w*, the device can be treated as a one-dimensional structure. For a certain doping concentration at the edge, the spatial distribution of the chemical potential μ and sheet carrier density *n* obtained from the simulation is shown in Figure 3c. Evidently, after the doping level was fixed at the edge, the doping profile decreased in proportion to the power function. For the “area-etching” and “perimeter-etching” devices, the top and bottom graphene flakes had the same shape, and the freshly etched edges overlapped each other, as shown in Figure 2c,d. In this case, the edges of the two graphene ribbons were assumed to overlap and have the same doping levels in the calculations. The simulation results of PVR as a function of *w* at *V*_g_ = 120 V are shown in Figure 3d (more simulations were performed to take into consideration other factors that may influence the PVR and are demonstrated in section 4 of the Supporting Information). Evidently, the PVR exhibited an approximately linear dependence on APR^–1^, which agrees with the experimental results shown in Figure 2g. A more detailed discussion will focus on the variations in the current densities and voltages at the peaks and valleys with decreasing APR and PVR. The output characteristics at *V*_g_ = 120 V of the simulated, “area-etching”, and “perimeter-etching” devices are shown in Figure 3e–g. The simulated output curves agreed closely with the experimental results. The results for all three devices showed that the current densities and voltages at the peaks and valleys decreased with decreasing APR. The significant decrease in the peak current density can be explained by the increasing area proportion affected by edge doping of the device. Additionally, the valley current, the voltage of the peak, and the voltage of the valley decreased. This phenomenon may be because the edge doping increased the sheet carrier density, which weakened the quantum capacitance effect, thereby resulting in a smaller separation of the energy alignment at the same *V*_g_. Thus, a lower *V*_d_ is required to achieve resonant tunneling conditions.

By harnessing the geometric dependence proposed in this study, a GRTD with a high PVR was presented. The output characteristics of device D at 300 K are shown in Figure 4a. This device had the highest APR of 3.87 μm in Figure 1g, which can effectively reduce the influence of edge doping. It exhibited the highest room-temperature PVR (14.9) among the 14 devices (an air stability test is demonstrated in section 5 of the Supporting Information). By reduction of the temperature to 9.6 K, the PVR further increased to 23.9, as shown in Figure 4b, because of the smaller doping profile. The output characteristics and PVR of device D at various temperatures are shown in Figure 4c,d. Evidently, the current densities and voltages at the peaks and valleys all decreased with increasing temperature, thereby exhibiting an effect similar to that of the APR in Figure 3f,g, which suggests that the temperature can also affect the PVR by adjusting the edge doping profile. Figure 4e shows a comparison of the PVR values obtained in this study to those reported in the literature. To the best of our knowledge, both the room-temperature and low-temperature PVR values obtained in this study are the highest ever reported for any 2D-material vertical NDR device. Optimization of the edge-doping profile can further improve the PVR, and the high PVR in this work may promote the future development of RTDs for applications such as ultrahigh frequency oscillators.

**Figure 4:**
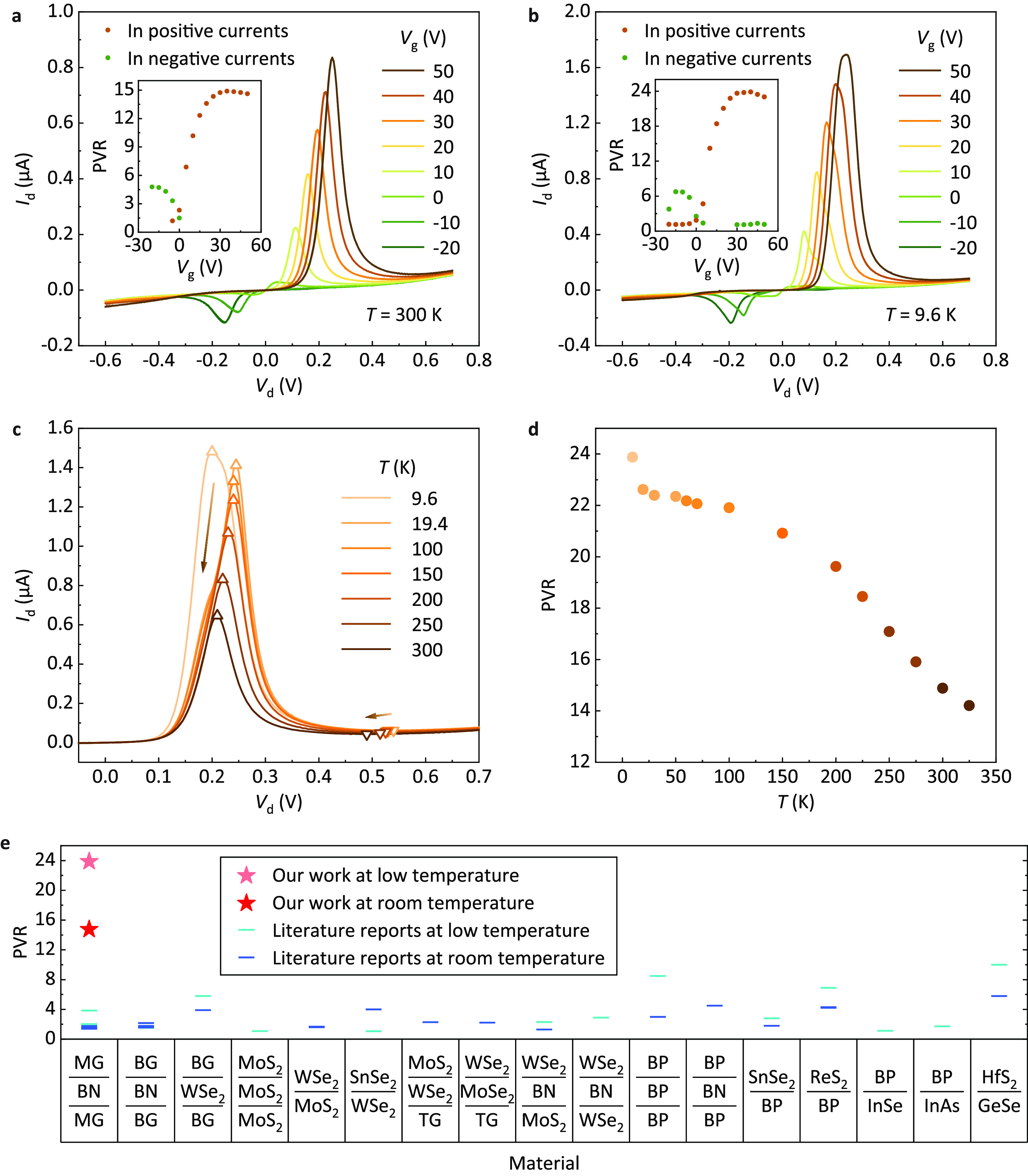
Toward high PVR. (a,b) Output characteristics of a high-PVR device (device D) under various gate voltages *V*_g_ at 300 K (a) and 9.6 K (b). The insets show extracted PVR values as a function of *V*_g_. (c) Output characteristics of the high-PVR device at various temperatures *T*. For each *T*, the output curve containing the highest PVR is chosen from different *V*_g_’s. Arrows show the directions that peaks and valleys change along with increasing *T*. (d) Extracted PVR values of the high-PVR device as a function of *T*. (e) A PVR comparison at room and low temperature between the high-PVR device in this study and 2D-material vertical NDR devices reported in the literature. References: MG (monolayer graphene)/BN (boron nitride)/MG,^13,19,23^ BG (bilayer graphene)/BN/BG,^18,20,21^ BG/WSe_2_/BG,^22^ MoS_2_/MoS_2_/MoS_2_ (stacked trilayer MoS_2_),^24^ MoS_2_/WSe_2_,^15,26^ WSe_2_/SnSe_2_,^28,34^ TG (trilayer graphene)/WSe_2_/MoS_2_,^25^ TG/MoSe_2_/WSe_2_,^25^ MoS_2_/BN/WSe_2_,^29^ WSe_2_/BN/WSe_2_,^31^ BP (black phosphorus)/BP/BP (twisted BP homostructure),^37^ BP/BN/BP,^36^ BP/SnSe_2_,^27^ BP/ReS_2_,^14,32^ InSe/BP,^35^ InAs/BP,^33^ GeSe/HfS_2_.^17^

In summary, the NDR characteristics of the RTD were found to be geometrically dependent. A new physical parameter, the effective permeation depth, λ_eff_, was introduced to take into account the edge doping. The results of the etching experiment, analytical model, and numerical simulation all agreed very well, and they confirmed that area-to-perimeter ratio is indeed key to PVR. Therefore, only devices with large area-to-perimeter ratios can have a high homogeneity for coherent resonant tunneling. Such a design rule allowed us to achieve record PVRs up to 14.9 at room temperature and 23.9 at low temperature. This approach can also be applied to other 2D-material-based RTDs and vdW heterostructure devices.

## Supporting Information Available

Methods, detailed information on 14 devices, numerical simulation, other factors that influence the PVR, and air stability of GRTD (PDF)

## Abbreviations

RTD: resonant tunneling diode
PVR: peak-to-valley ratio
vdW: van der Waals
2D: two-dimensional
NDR: negative differential resistance
h-BN: hexagonal boron nitride
GRTD: graphene resonant tunneling diode
APR: area-to-perimeter ratio
MG: monolayer graphene
BN: boron nitride
BG: bilayer graphene
TG: trilayer graphene
BP: black phosphorus
