# Supplementary material for: Toward High-Peak-to-Valley-Ratio Graphene Resonant Tunneling Diodes
Source: Nano Lett. 2023 Sep 5;23(17):8132–9. doi: 10.1021/acs.nanolett.3c02281 (PMC10510586; doi:10.1021/acs.nanolett.3c02281)
Supplement: Supplementary file 1 — nl3c02281_si_001.pdf [file nl3c02281_si_001.pdf]

Supporting Information for

Toward High-Peak-to-Valley-Ratio Graphene Resonant Tunneling Diodes

Zihao Zhang[1]‡, Baoqing Zhang[1]‡, Yiming Wang[1], Mingyang Wang[1], Yifei Zhang[1], Hu Li[1], Jiawei Zhang[1,2], Aimin Song[1,3]**

[1]Shandong Technology Center of Nanodevices and Integration, School of Microelectronics, Shandong University, Jinan, 250100, China.

[2]Suzhou Research Institute, Shandong University, Suzhou, 215123, China.

[3]Department of Electrical and Electronic Engineering, University of Manchester, Manchester, M13 9PL, United Kingdom.

‡These authors contributed equally.

*Correspondence to: Jiawei Zhang (E-mail: Jiawei.Zhang@sdu.edu.cn); Aimin Song (E-mail: A.Song@manchester.ac.uk).

1. Methods

Graphene and h-BN flakes were mechanically exfoliated from graphite crystals (NGS Naturgraphit) and h-BN crystals (2D Semiconductors) using adhesive tape (Nitto ELP BT-130E-SL). The top and bottom h-BN flakes were both tens of nanometres thick, whereas their surfaces were atomically flat. In the dry transfer process, the 'pick up' and 'tear and stack' techniques[1] were adopted to guarantee the momentum matching. The polydimethylsiloxane (PDMS)/polymethyl methacrylate (PMMA) film successively picked up h-BN, graphene, h-BN, graphene, and h-BN. The top and bottom graphene flakes were torn from one graphene flake. The exfoliation and transfer processes were performed under ambient conditions. The source and drain electrodes were defined by electron-beam lithography (Raith e-LiNE Plus). The h-BN flakes were etched using inductively coupled plasma (ICP; Oxford Instruments PlasmaPro 100 Cobra) to expose the graphene. The one-dimensional contacts[2] were formed by 3-nm Cr/70-nm Au, which was deposited by electron-beam evaporation (HHV auto 500).

The device was placed inside a cryogenic probe station (Lakeshore CRX-VF) for room-temperature and low-temperature measurements. The electrical measurements were performed using a Keysight B2902A instrument.

2. Detailed information on 14 devices

Table S1. Size information on 14 devices.

Device	Number of interlayer h-BN layers	Area (μm^2)	Perimeter (μm)	Highest PVR
Device #1	3	2.67	9.33	1.009
Device #2	3	9.75	13.4	1.18
Device #3	5	15.2	18.4	2.86
Device #4	4	51.9	33.9	3.77
Device #5	3	32.2	24.8	3.83
Device #6	4	25.3	26.0	4.09
Device #7 (Device B in the main text before etching)	4	30.6	22.7	4.30
Device #8	4	10.9	18.1	4.35
Device #9 (Device C in the main text before etching)	4	23.9	19.8	5.43
Device #10	5	25.0	26.9	6.12
Device #11	4	114.8	67.9	8.45
Device #12 (Device A in the main text)	4	49.0	32.2	9.02
Device #13	4	31.0	24.8	11.8
Device #14 (Device D in the main text)	5	390	101	14.7

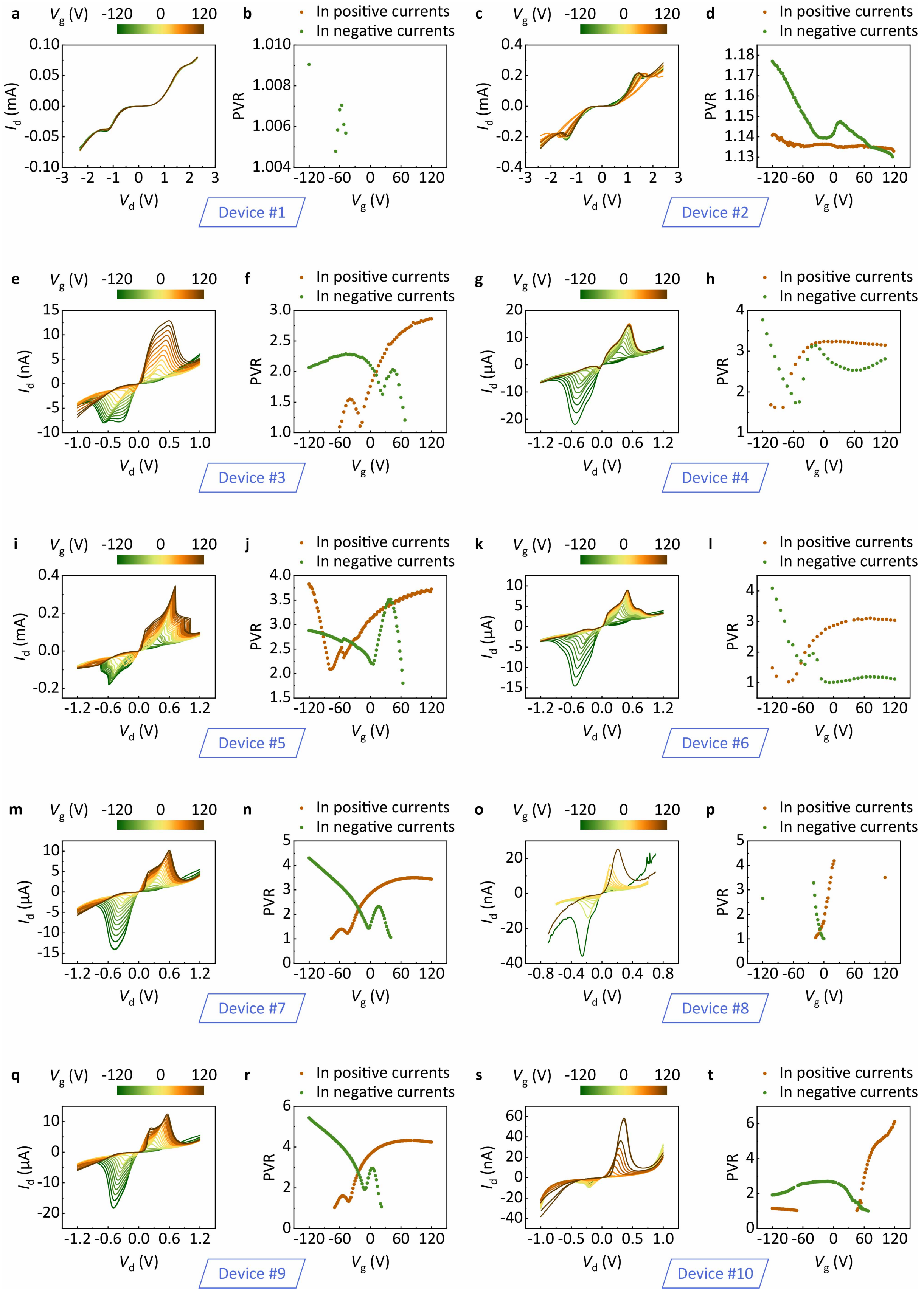
a
b
c
d
e
f
g
h
i
j
k
l
m
n
o
p
q
r
s
t
V_g (V) -120 0 120
In positive currents
In negative currents
I_d (mA)
I_d (nA)
I_d (μA)
V_d (V)
V_g (V)
PVR
Device #1
Device #2
Device #3
Device #4
Device #5
Device #6
Device #7
Device #8
Device #9
Device #10

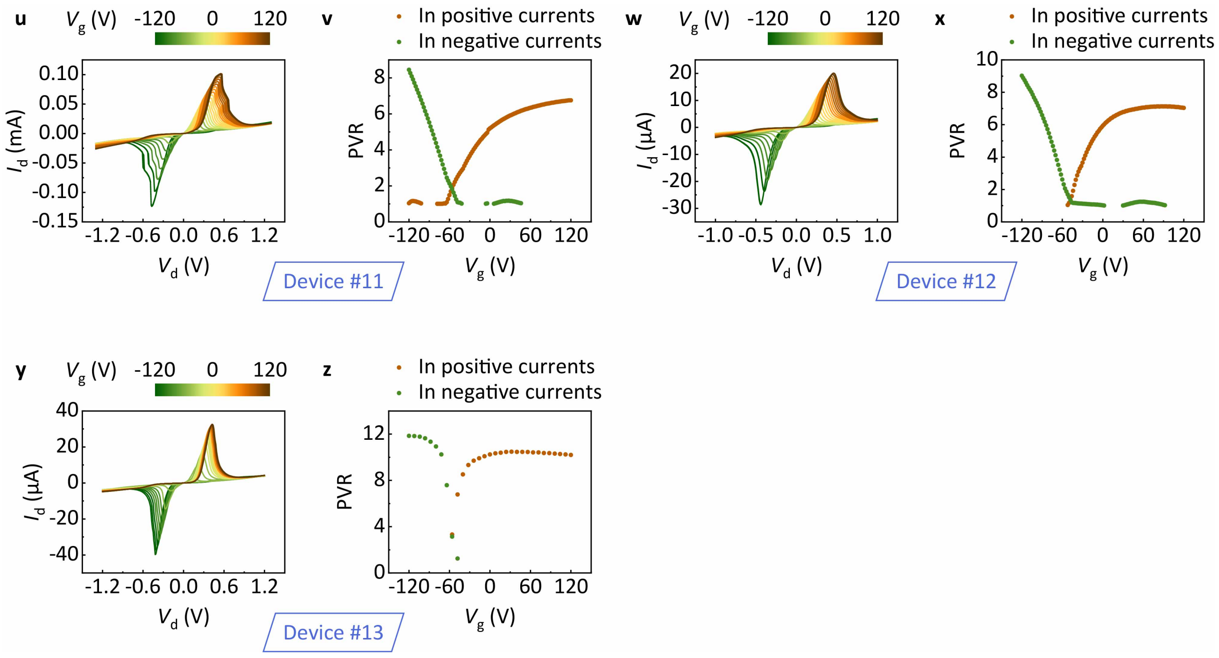

Figure S1. Output characteristics and extracted PVR values of 13 devices. (a–z) Output characteristics and PVR of Devices #1–#13.

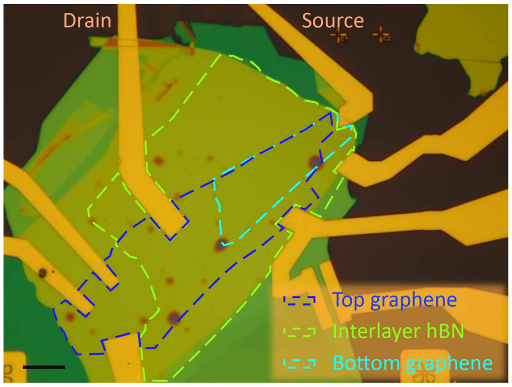

Figure S2. Optical micrographs of the high-PVR device (Device #14); the scale bar is 10 μm.

3. Numerical simulation

The numerical simulations in this study were based on the following principles. The drain current I_d of the GRTD is an integral of the tunnelling current density J_{tun} throughout the overlapping region of the two graphene flakes in real space (x, y) and is expressed as

$$I_d = \int J_{tun}(x, y)\, dx dy. \tag{S1}$$

For each coordinate pair (x, y), J_{tun} is an integral of the tunnelling throughout two Dirac cones in reciprocal space (k_x, k_y). Without considering a constant factor, it can be expressed as[3]

$$J_{tun}(x, y) = \int \frac{\gamma}{(s_T v_F |\boldsymbol{k}_T| - s_B v_F |\boldsymbol{k}_B| + \Delta\varphi)^2 + \gamma^2} (f_T - f_B)\, dk_x dk_y. \tag{S2}$$

Here, v_F is the Fermi velocity of graphene; γ is the broadening of electronic states; $s_{T/B}$, which has a value of 1 for the conduction band and −1 for the valence band, is the band index of the top or bottom graphene flake. $\boldsymbol{k}$ is the wave vector; $\boldsymbol{k}_{T/B}$ is the wave vector starting at the Dirac point of the top or bottom graphene flake; $\boldsymbol{K}_{T/B}$ is the wave vector of the Dirac point of the top or bottom graphene flake; thus, $\boldsymbol{k}$ is expressed as

$$\boldsymbol{k} = k_x \boldsymbol{l}_x + k_y \boldsymbol{l}_y = \boldsymbol{K}_T + \boldsymbol{k}_T = \boldsymbol{K}_B + \boldsymbol{k}_B. \tag{S3}$$

For a small twist angle θ, the wave vector difference of two Dirac points is approximately

$$\boldsymbol{K}_T - \boldsymbol{K}_B = \theta \boldsymbol{l}_z \times \boldsymbol{K}_T. \tag{S4}$$

Here, $\boldsymbol{l}_{x/y/z}$ is the unit vector of each direction in reciprocal space; $\Delta\varphi = \varphi_T - \varphi_B$ is the energy difference of two Dirac points. Electrostatic doping $n_{T/B,ele}$ and edge doping $n_{T/B,dop}$ are considered to contribute to the sheet carrier density of the top or bottom graphene flake $n_{T/B}$, which is counted positively for positive charge and is expressed as

$$n_{T/B} = n_{T/B,ele} + n_{T/B,dop}(x, y). \tag{S5}$$

Considering charge conservation, the sheet carrier density of the gate electrode n_g satisfies

$$n_{\mathrm{T,ele}} + n_{\mathrm{B,ele}} + n_{\mathrm{g}} = 0. \tag{S6}$$

The chemical potential of the top or bottom graphene flake $\mu_{\mathrm{T/B}}$ is given by

$$\mu_{\mathrm{T/B}} = -n_{\mathrm{T/B}}\hbar v_{\mathrm{F}}\sqrt{\pi/|n_{\mathrm{T/B}}|}. \tag{S7}$$

Considering a conservative field, without considering other resistances in the circuit, the drain voltage V_{d} satisfies

$$\mu_{\mathrm{T}} - \mu_{\mathrm{B}} + \Delta\varphi - eV_{\mathrm{d}} = 0. \tag{S8}$$

The gate charge corresponds to the electric field in the gate dielectric, and the gate voltage V_{g}, thickness of SiO_2 $d_{\mathrm{SiO_2}}$, and relative dielectric constant of SiO_2 $\varepsilon_{\mathrm{SiO_2}}$ satisfy

$$eV_{\mathrm{g}} = \frac{e^2 d_{\mathrm{SiO_2}} n_{\mathrm{g}}}{\varepsilon_0 \varepsilon_{\mathrm{SiO_2}}}. \tag{S9}$$

The electrostatic doping of the top graphene flake corresponds to the electric field in the interlayer h-BN flake, and the thickness of interlayer h-BN flake d_{BN}, number of interlayer h-BN layers l_{BN}, and relative dielectric constant of h-BN flake $\varepsilon_{\mathrm{BN}}$ satisfy

$$\Delta\varphi = -\frac{e^2 d_{\mathrm{BN}} n_{\mathrm{T,ele}}}{\varepsilon_0 \varepsilon_{\mathrm{BN}}}, \tag{S10}$$

$$d_{\mathrm{BN}} = 0.33\ \mathrm{nm} \times l_{\mathrm{BN}}. \tag{S11}$$

Equations (S3) and (S4) involve momentum matching, where θ is an important factor. Equations (S5)–(S11) involve energy matching. All unknowns can be obtained by solving the equations, where $n_{\mathrm{T/B,dop}}$ passes the inhomogeneous distribution in real space (x, y) to J_{tun}, thus leading to the broadening and suppression of the resonant tunnelling peak, which is the focus of this study. Moreover, the tunnelling process is determined by the difference in the number of quantum states occupied by the carriers between the top and bottom graphene flakes. $f_{\mathrm{T/B}}$ satisfies the Fermi–Dirac distribution and is given by

$$f_{\mathrm{T/B}} = \frac{1}{\exp\left(\frac{s_{\mathrm{T/B}} v_{\mathrm{F}} |\boldsymbol{k}_{\mathrm{T/B}}| - \mu_{\mathrm{T/B}}}{k_{\mathrm{Bol}} T}\right) + 1}. \tag{S12}$$

Here, k_{Bol} is the Boltzmann constant, and T is the temperature. The numerical simulation in Figure 3d used the parameters: $\gamma = 3$ to 15 meV; $\theta = 1°$; $l_{\mathrm{BN}} = 4$, which is the same as Devices B and C used in the etching experiment; $d_{\mathrm{SiO_2}} = 300$ nm; $V_{\mathrm{g}} = 120$ V; $T = 300$ K.

Two treatments exist for the distribution of edge doping $n_{\mathrm{T/B,dop}}$ (x, y): 'double-flake doping', which assumes that the two graphene flakes coincide in a location and have the same doping level, and 'single-flake doping', which assumes that one graphene flake has a finite size and some edge doping, whereas the other has an infinite size and no edge doping. Because the etching aligns the edges of the two graphene flakes, the etching experiment illustrated in Figure 2e–g should be closer to double-flake doping. Because the edges of the two graphene flakes rarely coincide in location after dry transfer, the situation of several devices in Figure 1e–g should be closer to single-flake doping. The general situation is somewhere between double- and single-flake doping. The simulation results of the PVR comparison between double- and single-flake doping are shown in Figure S3. The two treatments agree qualitatively, and thus do not affect the conclusion of this study. Quantitatively, single-flake doping decreases the PVR more significantly owing to the greater damage to device homogeneity. The numerical simulation results presented in Figure S3 used the parameters: $\gamma = 3$ to 15 meV; $\theta = 1°$; $l_{\mathrm{BN}} = 4$; $d_{\mathrm{SiO_2}} = 300$ nm; $V_{\mathrm{g}} = 120$ V; $T = 300$ K.

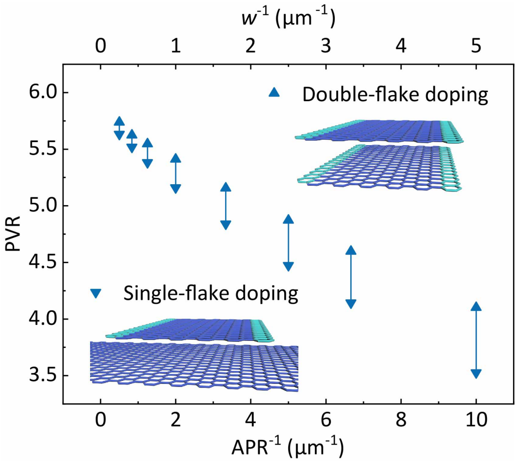

Figure S3. Comparison between double- and single-flake doping. Simulation results of PVR as a function of APR and the width *w*. For single-flake doping, suppose that the top graphene flake has a width *w* and the bottom graphene flake has an infinite width. The insets show the double- and single-flake doping schematics.

4. Other factors that influence the PVR

In addition to the APR metric emphasized in this paper, traditional factors reported in past literature,[1,3–6] such as the broadening of electronic states γ, twist angle θ, and number of interlayer h-BN layers l_{BN}, should also not be neglected. In the literature, the PVR was considered to decrease with increasing γ and θ, and l_{BN} influenced the tunnelling current density. Simulation results of the PVR as a function of γ, θ, and l_{BN} are shown in Figure S4. After considering various APRs, PVR still decreases with γ and θ; however, l_{BN} between 3 and 5 influences the PVR insignificantly. Therefore, in this study, γ was considered as an intrinsic property of materials that cannot be controlled, θ was minimized with the help of the 'tear and stack' technique, and l_{BN} was maintained between 3 and 5. The numerical simulation results presented in Figure S4 used the following parameters. In Figure S4a: $\theta = 1°$; $l_{BN} = 4$. In Figure S4b, $\gamma = 10$ meV; $l_{BN} = 4$. In Figure S4c: $\gamma = 3$ meV; $\theta = 1°$. In all the figures: $d_{SiO_2} = 300$ nm; $V_g = 120$ V; $T = 300$ K.

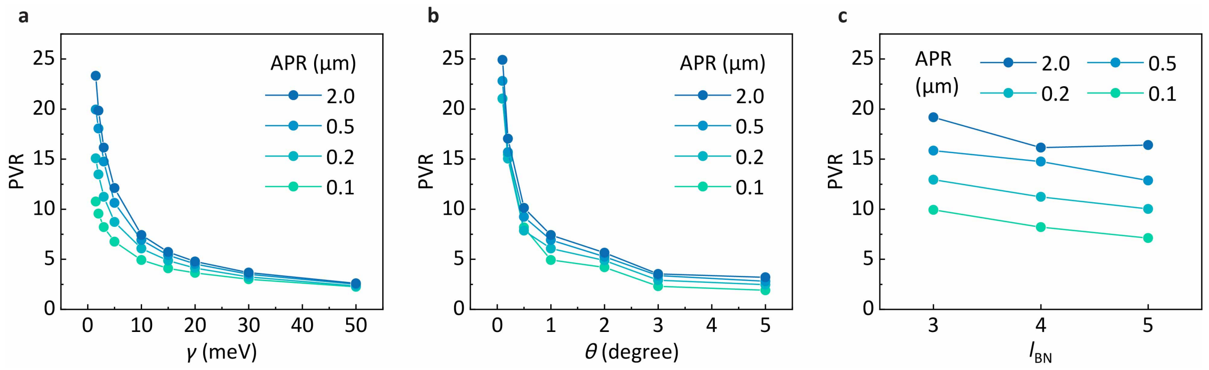

Figure S4. Influence of the broadening of electronic states γ, twist angle θ, and number of interlayer h-BN layers l_{BN} to the PVR. (a–c) Simulation results of PVR as a function of γ (a), θ (b), and l_{BN} (c) with various APRs.

5. Air stability of GRTD

Electronic devices are widely believed to lose their performance over time. In this study, this phenomenon also affected the reliability of the etching experiment. The electronic characteristics of the high-PVR device measured on 25 August, 2022, at room temperature are shown in Figure S5. These differ slightly from the results shown in Figure 4a, which were measured on 25 May, 2022. Essentially, the same device exhibited the highest PVRs of 14.3 and 14.9 3 months apart, thus demonstrating an acceptable air stability. The etching experiment in this study was performed within 1 month, from 21 March to 20 April, 2022, thus proving that the decrease in PVR in the etching experiment was not owing to long-term storage.

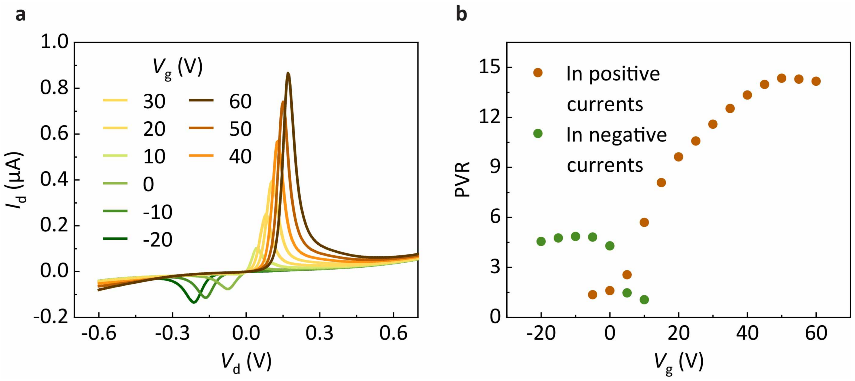

Figure S5. Electronic characteristics of the high-PVR device (Device D) from another measurement at room temperature to test its air stability. (a) Output characteristics. (b) PVR as a function of gate voltage V_g.

REFERENCES

1. Kim K, Yankowitz M, Fallahazad B, et al. van der Waals Heterostructures with High Accuracy Rotational Alignment. *Nano Lett.* **16**, 1989–1995 (2016).

2. L. Wang, I. Meric, P. Y. Huang, et al. One-Dimensional Electrical Contact to a Two-Dimensional Material. *Science* **342**, 614–617 (2013).

3. Mishchenko, A., Tu, J., Cao, Y. et al. Twist-controlled resonant tunnelling in graphene/boron nitride/graphene heterostructures. *Nat. Nanotechnol.* **9**, 808–813 (2014).

4. Fallahazad B, Lee K, Kang S, et al. Gate-tunable resonant tunneling in double bilayer graphene heterostructures. *Nano Lett.* **15**, 428–433 (2015).

5. Burg GW, Prasad N, Fallahazad B, et al. Coherent Interlayer Tunneling and Negative Differential Resistance with High Current Density in Double Bilayer Graphene-WSe_2 Heterostructures. *Nano Lett.* **17**, 3919–3925 (2017).

6. Kuzmina A, Parzefall M, Back P, et al. Resonant Light Emission from Graphene/Hexagonal Boron Nitride/Graphene Tunnel Junctions. *Nano Lett.* **21**, 8332–8339 (2021).
